# The POSE-2 Procedure for People with Obesity: A Safe and Effective Treatment Option

**DOI:** 10.1007/s11695-024-07488-8

**Published:** 2024-09-05

**Authors:** Marijn T. F. Jense, Tymen Hodde, Inge H. Palm-Meinders, Paul H. A. Bours, Khalida Soufidi, Evert-Jan G. Boerma, Jan Willem M. Greve

**Affiliations:** 1 Bariatric Surgery at Zuyderland Medical Center, Henri Dunantstraat 5, 6419 PC Heerlen, The Netherlands; 2 Dutch Obesity Clinic South, John F. Kennedylaan 301, 6419 XZ Heerlen, The Netherlands; 3 NUTRIM, Institute for Nutrition and Translational Research in Metabolism, Maastricht, The Netherlands; 4 Maastricht University, Maastricht, The Netherlands; 5 Gastro-Enterology at Zuyderland Medical Center, Henri Dunantstraat 5, 6419 PC Heerlen, The Netherlands; 6 Department of Surgery, NUTRIM, Institute for Nutrition and Translational Research in Metabolism, Maastricht University Medical Center, Maastricht, The Netherlands

**Keywords:** POSE-2 procedure, Bariatric treatment, Lifestyle intervention, Obesity, Weight loss, Endoscopic treatment

## Abstract

**Purpose:**

Besides lifestyle interventions, medication, and surgery, endoscopic options are becoming part of the current treatment landscape for people with obesity. With the POSE (Primary Obesity Surgery Endoscopic) procedure, endoluminal folds are created in the stomach with full-thickness sutures. Recently, the modified version, POSE-2, was introduced in clinical practice. This study aims to evaluate the safety and effectiveness of the POSE-2 procedure after one year in patients with obesity.

**Materials and Methods:**

All patients treated with the POSE-2 procedure between March 2019 and November 2022 in the Zuyderland Medical Center and the Dutch Obesity Clinic were included in this retrospective data study. Inclusion criteria are as follows: age between 18 and 65 years and a BMI > 30 kg/m^2^. All patients with contraindications for the POSE-2 procedure were excluded.

**Results:**

Forty-nine patients were included of which 86% were female, with a mean age of 46 years and mean BMI of 34.6 kg/m^2^. Total weight loss was evaluated at 3, 6, and 12 months and was 11.5%, 13.2%, and 14.8%, respectively. A median of 14 anchor sutures was used in a median procedure time of 50 min. All patients except one had same day discharge. Postprocedural complaints were mild and consisted of nausea and vomiting (36.7%) and pain (54.2%). No complications were recorded in this group. One week postprocedure, most patients (95.9%) reported feeling satisfied between meals.

**Conclusion:**

The POSE-2 procedure can be applied as a safe and effective treatment for people with obesity. This study presents a positive effect on weight reduction and no complications after 1 year of follow-up.

**Graphical Abstract:**

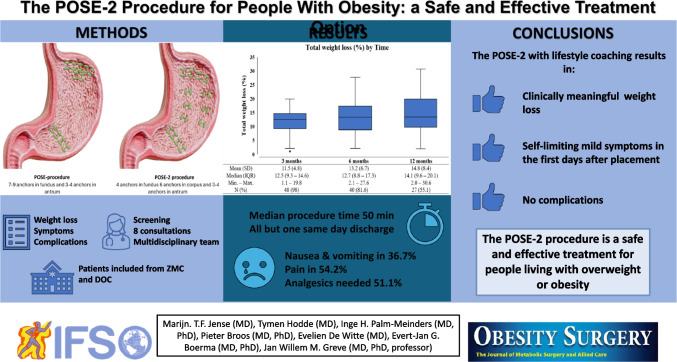

## Introduction

The range of treatment options for people with overweight and obesity is growing. Besides lifestyle interventions and surgery, non-surgical interventions such as anti-obesity medication and endoscopic options are becoming parts of the current treatment options, with promising results [1–3].

One of the endoscopic treatment options is the POSE (Primary Obesity Surgery Endoscopic) procedure. With the POSE procedure, endoluminal folds are created in the stomach wall with full-thickness sutures. As a consequence, the stomach size decreases which causes earlier satiety leading to a reduction in food intake and with that weight loss. Since this is an endoscopic procedure, it is less invasive compared to surgery [4, 5].

More recently, the modified version, the POSE-2 procedure, was introduced in clinical practice. With this procedure, a different combination and location of the sutures is used. During the POSE procedure, plications are solely made in the fundus of the stomach; during the POSE-2, plications are mainly made in the corpus and antrum. The end result of the POSE-2 is more similar to the gastric sleeve and aims to obtain more reduction of the stomach size and with that earlier satiety. Thus far, not many studies have described the effect of the POSE-2 procedure [6–8].

Therefore, this study aims to evaluate the effect of the POSE-2 procedure on weight loss in patients with obesity after 1 year. As secondary outcomes, this study aims to evaluate the complication rates and postprocedural symptoms.

## Methods

### Study Design

All data was retrospectively collected from the electronic patient files of the Zuyderland Medical Center (ZMC) and the Dutch Obesity Clinic (DOC). All patients have given their consent to use patient data for scientific research. For this data study, local approval was given by the local ethics committee in accordance with the ethical standards as laid down in the 2013 Declaration of Helsinki.

### Participant and Eligibility Criteria

All consecutive adult patients who received the POSE-2 procedure with additional lifestyle coaching between March 2019 and November 2022 in the ZMC were included in this study. Exclusion criteria for the POSE-2 procedure, and therefore in this study, were as follows: esophageal abnormalities (webs, strictures, diverticula), having a history of motility disorders of the esophagus or stomach, previous surgeries on the esophagus, stomach or intestines, moderate to severe reflux disease, severe gastroparesis, peptic ulcer, hiatal hernia > 3 cm, current pregnancy or lactation, use of corticosteroids, inflammatory gastrointestinal diseases (Crohn’s disease, ulcerative colitis), and impairing psychopathology. Patients with a BMI lower than 30 kg/m^2^ or higher than 40 kg/m^2^, liver cirrhosis or insufficiency, portal hypertension, severe coagulation disorders, inability to stop anticoagulants, current smokers, or patients who quit smoking < 6 weeks prior to screening were first discussed with a bariatric surgeon for eligibility. All eligibility criteria were obtained by questioning the patients about there medical history and by the endoscopy performed prior to the procedure.

### POSE-2 Procedure

All POSE-2 procedures were performed by a surgeon and a gastroenterologist under general anesthesia with the patient in the prone position. The surgeon had previous experience with the POSE procedure and followed training for the POSE-2 procedure [9]. Cefazolin/metronidazole 2000 mg/500 mg was given as prophylactic antibiotics. Before the procedure, the stomach was endoscopically checked for possible abnormalities or contraindications for the procedure (hiatal hernia, stenosis, peptic ulcer, etc.). For insufflation, carbon dioxide gas was used. The plications were created as follows: one row of four anchors in the antrum to create the “belt.” This reduces the circumference of the stomach and might decrease the gastric-emptying rate by creating and obstacle for food. Next, two rows of three anchors each were placed in the corpus, called the “suspender.” This narrows the gastric body. Lastly, one row of four anchors was placed below the fundus to further decrease the length of the stomach. This plication pattern is aimed to reduce the length and width of the stomach and differs from the original POSE procedure (Appendix Fig. 1).

### Postprocedure Protocol

After the procedure, patients were discharged on the same day if no complications occurred. Dietary advise was given, consisting of two phases: the liquid and solid food phase. In the first 2 weeks postprocedure, solely liquid foods were allowed, starting with clear liquids and building up to thicker liquid foods. The solid food phase started with soft solid foods which could be extended to firmer foods if tolerated. The recommended fluid intake was 1500–2000 ml a day. To prevent possible reflux symptoms, all patients received proton pump inhibitors during the first 6–12 weeks. If reflux symptoms persisted, the medication could be continued for a longer period.

### Follow-Up Lifestyle Intervention

Patients were followed up by the DOC for 1 year by enrolment in a multidisciplinary lifestyle intervention program. During this 1 year period, they had eight follow-up appointments. These were planned 1 day, 1 week, 4 weeks, 9 weeks, 3 months, 6 months, 9 months, and 12 months after the procedure and consisted of physical appointments and sometimes consultations by phone or video call. When a patient did not show up and did not respond to multiple consultations, they were considered loss to follow-up.

### Outcomes

The following parameters were recorded in this study: age, sex, preoperative weight, BMI, comorbidities, postoperative symptoms, compliance to protocol, and postoperative weight at 3, 6, and 12 months. The primary outcome measure is percentage of total weight loss (%TWL). Other additional weight parameters such as % excessive weight loss (EWL) were also recorded.

Effectiveness was determined using the minimum threshold for endoscopic bariatric and metabolic therapies (EBMTs) as established by the American Society for Gastrointestinal Endoscopy (ASGE)/American Society for Metabolic and Bariatric Surgery (ASMBS). This threshold is at least 25% of EWL measured at 12 months for people with obesity class II/III (BMI > 35 kg/m^2^) and an incidence of ≤ 5% for serious adverse events [10, 11]. Complications were scored using the Clavien-Dindo classification [12].

### Statistical Analysis

Statistical analysis was performed using IBM SPSS statistics version 26. The normality of all variables was assessed using the Shapiro–Wilk test. Variables with a normal distribution were displayed as mean with a standard deviation (SD). Variables with an abnormal distribution were displayed as median and interquartile range (IQR).

Dichotomous variables, such as patient characteristics, were analyzed using a frequency analysis. The primary outcome measure (%TWL) is a continuous variable and was analyzed using descriptive statistics.

To identify potential predictors of weight loss, the study group was divided into patients with a low %TWL, determined as < 15% TWL, and patients with a high %TWL, determined as ≥ 15% TWL. The groups were analyzed using a univariable logistical regression, expressed as an odds ratio (OR) with 95% confidence interval (95% CI). The variables age, sex, BMI, operation time, and number of Snowshoe Anchors were compared. If there were statistically significant differences within variables, a multivariable logistical regression was performed to rule out possible bias. An additional univariable and multivariable linear regression model was applied to the variables mentioned earlier using the %TWL as dependent continuous variable to rule out possible bias caused by the cutoff point. All variables were checked for non-linearity using a scatterplot. For all statistical analyses, *p* < 0.05 was considered statistically significant.

## Results

### Baseline Characteristics

Between March 2019 and November 2022, a total of 49 patients underwent the POSE-2 procedure in the ZMC and were included in this study. The study population consisted of 42 females and 7 males, with a mean age of 46 years and a mean BMI of 34.56 kg/m^2^. All comorbidities and further baseline characteristics are presented in Appendix Table 1.

### Procedure Characteristics

The median procedure time was 50 min (IQR 43–55) with procedure times ranging from 26 up to 156 min. The median number of Snowshoe Anchors was 14.0 (IQR 14–14) with the amount ranging from 10 to 16 Snowshoe Anchors. This number was established intraoperatively to attain the desired reduction in stomach size.

The median length of stay in the hospital was 1.0 day. One patient was kept overnight for observation due to a prolonged operating time caused by technical difficulties with the instruments. This patient was discharged the next day. All other patients were discharged from the hospital on the same day as the procedure.

### Postprocedural Symptoms and Compliance

In the first week after the procedure, 18 patients (36.7%) experienced mild nausea and/or vomiting, and 26 patients (54.2%) experienced pain of which 23 patients (88.5%) required the use of analgesics. Forty-seven patients (95.9%) reported feeling satisfied between meals. The mean number of days before patients returned to work was 7 (SD 4). Depending on outcome measure and time point, different numbers of data were missing, ranging from 0 to 30 missing values (Appendix Table 2).

The compliance for intake of medication and supplements was also recorded. Forty-three (89.6%) patients used their proton pump inhibitor (PPI) and multivitamin supplements daily, 31 (86.1%) patients their protein supplements, 45 (91.1%) patients had a regular eating pattern and fluid intake conform protocol, and no patients reported to use alcohol in the first week after the procedure.

### Weight Loss

Weight loss was assessed on 3, 6, and 12 months after the procedure. In the first 3 months, patients had a mean %TWL of 11.5 (SD 4.8). After 6 months, the mean %TWL was 13.2 (SD 6.7). After 12 months, the mean %TWL was 14.8 (SD 8.4). During this follow-up time, several patients were lost to follow-up, ranging from 1 patient (2%) on the first follow-up moment to 22 patients (44.9%) on the last follow-up moment (Appendix Fig. 2).

The mean %EWL at 3, 6, and 12 months was 44.7 (± 21.9), 50.9 (± 28.5), and 54.3 (± 35.4), respectively (Appendix Table 3).

### Predictors of High Weight Loss

After dividing the patients into low vs. high weight loss at 3 months follow-up, the low weight loss group consisted of 38 (79.2%) patients and the high weight loss group of 10 (20.8%) patients. The analysis was solely performed using the 3-month data since there were too many patients missing at later timepoints to perform statistically correct analyses. Exploring the possible factors associated with low vs. high weight loss, no statistically significant results were found using the logistic regression as described in the methods section. Multivariate logistical regression gave no further insights. Additional linear regression models using the %TWL at 3 months, correcting for the variables as mentioned in the methods section, also demonstrated no variables to be significant predictors of high weight loss in the present study.

### Complications

No readmissions, major bleeding, or any other complications occurred in this study group. No patients required an additional endoscopic or surgical intervention.

## Discussion

### Weight Loss

This study’s results demonstrate that the POSE-2 procedure is an effective treatment option with a mean %TWL after 12 months of 14.8 (SD 8.4). This weight loss result is in line with earlier studies regarding the POSE-2 procedure, which recorded a mean %TWL after 12 months of 15.7 (SD 6.8) and 17.8 (SD 9.5) [6, 8]. Regarding the threshold for EBMTs, one can determine this treatment as effective.

### Complications

Besides effectiveness, safety is an important characteristic of a treatment option. The current study shows that the POSE-2 procedure did not lead to any complications in our center and resulted in relatively mild post procedural complaints. In their prospective study, Lopez-Nava et al. described four patients (5%) with complications, two intra-operative gastric perforations, and two patients with asymptomatic hemoglobin drop within 24 h. All patients recovered completely [6]. Regarding the threshold for EBMTs, one can determine this treatment as safe.

### Postprocedural Symptoms

After the POSE-2 procedure, 36.7% of patients experienced nausea and/or vomiting, and 54.2% experienced pain of which 88.5% required the use of analgesics. This is consistent with the results of other studies [2, 6]. These symptoms all resolved within a week.

### Comorbidities

Due to the retrospective nature of this study, it was not possible to assess the comorbidities over time, since this was not a standard part of the follow-up. The number of patients in this study who suffered from comorbidities at baseline ranged from 2 to 16% depending on the comorbidity. This relatively low number of comorbidities might be due to the fact that the majority of patients had obesity class I or II. The prevalence of comorbidities in patients with obesity class III is higher [13].

Other studies have established the positive effect weight loss has on comorbidities for patients after the POSE-2 procedure. For instance, Al Khatry et al. present a significant improvement in patients with non-alcoholic fatty liver disease 12 months after the POSE-2 procedure, compared to patients who received lifestyle coaching only. Furthermore, Espinós et al. show an improvement of glucose homeostasis and satiation peptide response 6 months after POSE procedure [2, 8, 14, 15]. Taking this into account, one can expect positive effects on comorbidities when a reduction in weight is achieved.

### Durability

A possible influence on weight loss maintenance is the durability of the Snowshoe Anchors. Unfortunately, in the current study, no further research about durability was performed. However, this was investigated after 1 year using 15 patient cases in the study of Lopez et al. [6]. This study showed that all plications were intact, and no pathologic lesions were found. Only two patients had distension of the stomach; this might have contributed to their relatively low %TWL.

Lopez et al. further assessed durability of the plications in 26 patients after 24 months using gastroscopy and radiographic contrast in a prospective study. Plications were normal and the integrity of the gastric reduction was maintained [8]. Since these are only small studies, it would be interesting to investigate the durability of the POSE-2 procedure on a larger scale.

### Place in Treatment Landscape

The role of POSE-2 in the bariatric treatment landscape occupies a position between medication and surgery. Multiple studies have shown that once medication is stopped, most of the lost weight will reoccur [16]. On the other hand, surgery does present a durable effect, but with more risk of complications [17]. POSE-2 stands out as a less invasive alternative with the potential for long term effectiveness. This makes it a compelling choice for individuals living with obesity, particularly those hesitant to embrace surgery or facing challenges in maintaining long-term compliance with medication regimens. POSE-2 therefore offers a middle ground that combines effectiveness with reduced invasiveness, catering to the preferences and concerns of a specific patient population [18].

### Limitations of Study and Future Perspectives

The retrospective nature of the current study proved to be a limitation, since no information on comorbidity development or quality of life was part of the regular follow-up and was therefore not part of this study. Even though this was a retrospective study, the follow-up rate at one year was acceptable (55.1%).

Furthermore, the follow-up duration of 1 year does not show the long-term viability of the POSE-2 procedure. Future studies on the POSE-2 procedure should therefore focus on longer-term follow-up from at least 2, but preferably 5 years.

## Conclusion

The current study presents a positive effect on weight reduction and no complications during the procedure or until 1 year of follow-up. Therefore, we conclude that the POSE-2 procedure can be used as a safe and effective treatment for people with obesity.

## Figures and Tables

**Fig. 1:**
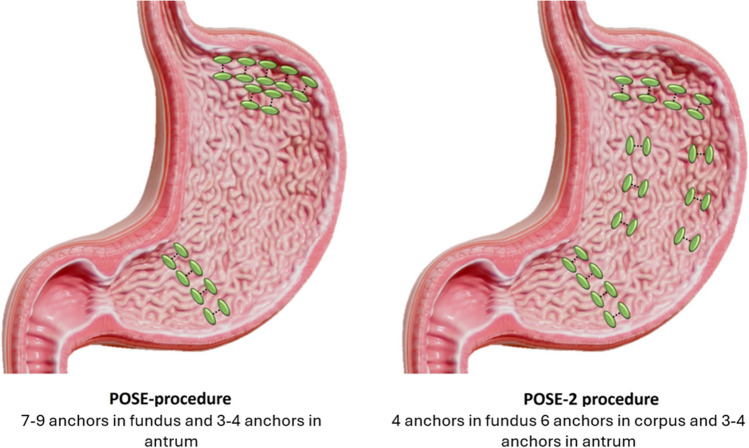
Difference in plication pattern between the original POSE-procedure and the POSE-2 procedure

**Fig. 2:**
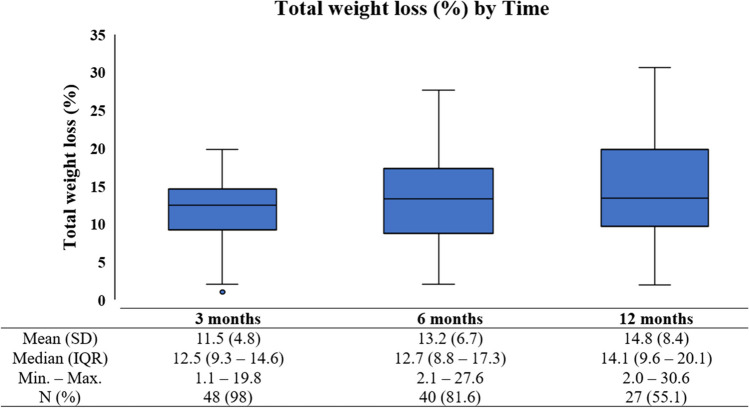
Boxplot presenting the %TWL of patients during the 12-month follow-up

## Data Availability

The data used to support the findings of this study are included within the article.

## Appendix

**Table 1:** Patient characteristics of the POSE-2 patient group

Patient characteristic	Count of *N* = 49(%)	SD	Minimum–maximum value
Sex	42 female (85.7)	-	-
Mean age (years)	46	9.00	26–63
Mean BMI (kg/m^2^)	34.56	3.07	29.13–41.42
Obesity class I Obesity class II Obesity class III	28 (57.1) 18 (36.7) 3 (6.1)	- - -	- - -
Hypertension	6 (12.2)		
Diabetes mellitus	1 (2)	-	-
OSAS	3 (6.1)	-	-
Hypercholesterolemia	2 (4.1)	-	-
Pyrosis	7 (14.3)	-	-
Active smokers	2 (4.1)	-	-

*SD* standard deviation, *cm* centimeters, *kg* kilograms, *m*^*2*^ square meters, *OSAS* obstructive sleep apnea syndrome

**Table 2:** Symptoms experienced and compliance in the first week after the procedure

Postprocedural symptom	Count	N%	Missing
Nausea/vomiting	18/49	36.7	0
Pain	26/48	54.2	1
Analgesics needed	23/45	51.1	4
Daily use of PPI	43/48	89.6	1
Multivitamin supplements used	43/47	91.5	2
Protein supplements used	31/36	86.1	13
Diet conform protocol	46/48	95.8	1
Regular eating pattern	45/47	95.7	2
Fluid intake conform protocol	45/49	91.1	0
Use of alcohol in first week	0/49	0	1
Satisfied between meals	47/49	95.9	0
Mean days to return to work (SD)	7 (4)	-	30

*PPI* proton pump inhibitor, *SD* standard deviation

**Table 3:** Excess weight loss percentage of patients during 12-month follow-up period

	%EWL on 3 months (*n* = 48)	%EWL on 6 months (*n* = 40)	%EWL on 12 months (*n* = 27)
Mean	44.65	50.85	54.33
Standard deviation	21.86	28.51	35.35
Min.–max. value	3.1–96.7	6.1–138.7	6.1–148.7

*EWL* excess weight loss
